# Roxadustat Versus Erythropoietin: The Comparison of Efficacy in Reversing Ventricular Remodeling in Dialysis Patients with Anaemia

**DOI:** 10.7150/ijms.87870

**Published:** 2024-02-12

**Authors:** Meng Ying Wang, Xiao Qi Liu, Ting Ting Jiang, Wen Tao Liu, Yang Huang, Yu Lin Huang, Feng Yong Jin, Qing Zhao, Qin Yi Wu, Gui Hua Wang, Xiong Zhong Ruan, Kun Ling Ma

**Affiliations:** 1 Institute of Nephrology, Zhongda Hospital, School of Medicine, Southeast University, Nanjing, 210009, China.; 2 John Moorhead Research Laboratory, Department of Renal Medicine, University College London (UCL) Medical School, Royal Free Campus, London, NW3 2PF, UK.; 3 Department of Nephrology, the Second Affiliated Hospital, School of Medicine, Zhejiang University, Hangzhou, 310003, China.

**Keywords:** anaemia, roxadustat, erythropoietin, ventricular remodeling, dialysis

## Abstract

**Background:** Renal anaemia and left ventricular hypertrophy are the main complications of chronic kidney disease and are shared among dialysis patients. This retrospective study aimed to compare the efficacies of the hypoxia-inducible factor prolyl hydroxylase inhibitor roxadustat and recombinant human erythropoietin in reversing ventricular remodeling in dialysis patients with renal anaemia.

**Methods:** A total of 204 participants underwent baseline examinations, including echocardiograms and laboratory tests, before being administered either treatment for at least 24 weeks from January 2018 to October 2021, after which follow-up examinations were conducted at 6 months. Propensity score matching based on key variables included age, gender, cardiovascular diseases, cardiovascular medications, dialysis course and the vascular access at baseline was performed to include populations with similar characteristics between groups.

**Results:** In total, 136 patients were included with roxadustat or recombinant human erythropoietin. The left ventricular mass index after treatment with roxadustat and recombinant human erythropoietin both significantly decreased after 6 months, but there was no significant difference in the change in left ventricular mass index between the two groups. In addition, the left ventricular end-diastolic diameters and left ventricular wall thickness, systolic blood pressure, and diastolic blood pressure significantly decreased in the roxadustat group. Roxadustat and recombinant human erythropoietin also increased haemoglobin significantly, but there was no significant difference in the change in haemoglobin between the two groups. The results of multiple linear regression showed that the change in haemoglobin was independent factor affecting the improvement of left ventricular mass index.

**Conclusions:** The increase of haemoglobin was associated with improving left ventricular hypertrophy in dialysis patients. However, the beneficial effects between roxadustat and recombinant human erythropoietin on left ventricular mass index did not show clear superiority or inferiority in six months

## Introduction

As one of the most common complications of chronic kidney disease (CKD), cardiovascular disease (CVD) is also one of the main causes of death in patients with CKD 1. CVD in these patients is related to many risk factors, such as hypertension, diabetes, dyslipidemia, anaemia, and abnormal calcium and phosphorus metabolism associated with uremia. Furthermore, cardiac remodeling, characterized by left ventricular hypertrophy (LVH) and myocardial fibrosis, is common in CKD patients. Studies have indicated that patients undergoing long-term renal replacement therapy have a high incidence of LVH^2^, ^3^. Increased in left ventricular volume is independently associated with CVD incidence and mortality in end-stage renal disease (ESRD) patients^4^, ^5^. It was previously reported that a decrease in the concentration of haemoglobin (Hb) by 1 g/dL resulted in an 18% increase in the risk of heart failure in patients on dialysis 6. Renal anaemia in patients with CKD can lead to a reduced myocardial oxygen supply, resulting in increased myocardial cell necrosis and apoptosis, which induces oxidative stress, activating various inflammatory pathways and the sympathetic nervous system and ultimately leading to pathological cardiac remodeling^7^.

Erythrocyte-forming stimulants (ESA) have long been the primary treatment option for patients with renal anaemia and improve the quality of life in CKD patients receiving dialysis and non-dialysis treatment and reduce the incidence of anaemia-related CVD and the need for blood transfusion^8^. According to Parfrey's meta-analysis^9^, in patients with severe anaemia before treatment (at baseline) (defined as a concentration of Hb < 10g/dL, with the average baseline Hb concentration in each study as low as 5.9 g/dL), increasing the concentration of Hb to ≤ 12g/dL by ESA could significantly reduce the left ventricular mass index (LVMI). One study comparing the baseline and post-renal transplantation echocardiographs in 232 patients with ESRD showed that patients with left ventricular dysfunction had improved cardiac structure and function and higher levels of Hb after renal transplantation compared to pre-renal transplantation^10^. Therefore, improving the level of Hb may be effective for reversing cardiac remodeling and cardiovascular prognosis.

Despite the clinical significance of ESA for the treatment of renal anaemia, some studies confirmed that supraphysiological doses of ESA were associated with an increased risk of cardiovascular events, CKD progression, vascular pathway thrombosis, and mortality. One study in nephrectomized mice recently showed that hypoxia-inducible factor prolyl hydroxylase inhibitors (HIF-PHIs) reduced myocardial hypertrophy and fibrosis in these mice by restoring capillary density and improving mitochondrial morphology^11^. HIF-PHIs are a novel class of orally available drugs that have been associated with a lower risk of cardiovascular events in dialysis patients while improving renal anaemia^12^. Nevertheless, to date, the ability of HIF-PHIs to aid in the reversal of cardiac remodeling in dialysis patients with renal anaemia has not been explored. Therefore, this research aims at probing and comparing the clinical efficacy of two different drugs for renal anaemia (the HIF-PHI roxadustat and ESA) in ventricular remodeling in dialysis patients with renal anaemia through clinical retrospective analysis.

## Methods

### Study population and study design

The hospital's electronic medical record data of dialysis patients receiving either oral roxadustat or parenteral ESA three times per week for 6 months were retrospectively reviewed in a single tertiary healthcare centre (Department of Nephrology, Zhongda Hospital Affiliated to Southeast University, Nanjing, China) between January 2018 and October 2021. All patients had pre- and post-treatment echocardiograms at six months. Patients (age > 18 years) in stage 5 CKD who were previously diagnosed with renal anaemia (Hb < 100g/L) and began long-term haemodialysis (HD) (dialysis time ≥ 12 weeks) were eligible for the study. Exclusion criteria for the current analysis included: 1 any clinically significant or active underlying infection; 2 chronic liver disease; 3 acute coronary syndrome or thromboembolic event within 1 year prior to medication; 4 anaemia is caused by chronic inflammatory diseases (e.g. systemic lupus erythematosus and rheumatoid arthritis) or hematological diseases (e.g. myelodysplastic syndrome and multiple myeloma), or other non-renal anaemia; 5 blood transfusion within 12 weeks of study or expected need for blood transfusion; 6 poor long-term blood pressure control; and 7 congenital heart disease, valve disease, rheumatic heart disease, or other heart diseases that result in potential changes to heart structure and/or function. All eligible dialysis patients with renal anaemia in the electronic medical record system were reviewed. Eligible patients were divided into roxadustat and rhEPO groups based on treatment, with oral roxadustat three times weekly (TIW), or ESAs administered intravenously or subcutaneously. In roxadustat group, for ESA-untreated patients, the starting dose was 100mg for patients weighing 45-60kg or 120mg for patients weighing ≥60kg. For patients who previously received ESA, the starting dose was 70-120 mg TIW based on the previous ESA dose. In rhEPO group, patients treated with ESA were dosed according to the ESA dose before entering the study, and ESA-naive patients were dosed according to 50 IU/kg TIW, with the dose adjusted based on local prescribing information.

Information related to the demographics, dialysis-related medical history, medications currently being taken, comorbidities, blood pressure, echocardiogram, and laboratory data of the patients in the study were collected directly from the patients' electronic medical records. All patients were evaluated before treatment (baseline) and 6 months after treatment. The manuscript was prepared based on the Strengthening the Reporting of Observational Studies in Epidemiology (STROBE) (13)statement. The Ethics Committee approved the study of Zhongda Hospital, Southeast University. The number of the ethics certificate is 2018ZDSYLL093-P01. The study was performed in accordance with the declaration of Helsinki. The data were anonymous, and the requirement for informed consent was waived.

Anaemia is diagnosed in adults with CKD when the Hb concentration is < 13.0 g/dL (< 130 g/L) in males and < 12.0 g/dL (< 120 g/L) in females based on the Kidney Disease - Improving Global Outcomes (KDIGO) guidelines^14^. Hypertension is defined as systolic blood pressure (SBP) higher than 130 or a diastolic blood pressure (DBP) higher than 80 mm Hg based on the American College of Cardiology/American Heart Association (ACC/AHA) guidelines^15^. Left ventricular (LV) parameters obtained by 2D echocardiography are employed to assess the estimated left ventricular mass. The following LVH evaluation criteria based on LV wall thickness and cavity dimensions of the LV were published by the American Society of Echocardiography (ASE) in conjunction with the European Society of Echocardiography^16^: estimated LVMI > 103 g/m^2^ for males and > 89 g/m^2^ for females is abnormal. LVH was defined as LVMI> 115g/m^2^ in males and LVMI> 95g/m^2^ in females. Severe LVH was defined as LVMI>148 g/m^2^ in males and LVMI>121g/m^2^ in females.

### Measurements and calculations

The primary outcome of this study was the changes in LVMI, left ventricular ejection fraction (LVEF), left ventricular end-diastolic diameters (LVEDd), interventricular septal thickness (IVST), and left ventricular wall thickness (LVPWT) over 6 months after initiation of the treatment interventions. All patients were examined by Philips IE33 echocardiography diagnostic instrument and S5-1 heart probe with a frequency of 3.5-5mhz in a Core Echo Laboratory (Division of Cardiology, Zhongda Hospital Affiliated to Southeast University, Nanjing, China), to ensure consistent measurement methodology. Patients were placed in the supine position or left decubital position. Measurements were performed on three to five consecutive beats, from which the mean values were calculated. All readings were taken by qualified technicians and subsequently reviewed by senior echocardiographers. These changes were assessed by echocardiographic imaging by performing the biplane method of disk summation (modified Simpson's rule) according to the recommendations by the ASE. The LVMI was calculated using the following formula:

*LVMI = LVM/body surface area (BSA)* (1)

in which the Left Ventricular Mass (LVM) was a calculated based on the following formula:

*LVM = 0.8 × (1.04 × [IVST + LVPWT + LVEDd]^3^ - [LVEDd^3^]) + 0.6* (2)

### Propensity score matching

Propensity score matching was performed on the two cohorts to minimize the deviation caused by confounding factors between patients treated with roxadustat and rhEPO in the dialysis population. Matching based on clinical covariates considered to impact on the patient's outcomes, including age, gender, cardiovascular diseases, cardiovascular medications, dialysis course and the vascular access, was employed using 1:1 nearest neighbor algorithm with a caliper value of 0.1.

### Statistical analysis

The Shapiro-Wilk test was adopted to assess the normality of continuous variables. The missing variables conforming to the normal distribution were filled with the mean, otherwise filled with the median. Variables of normal distribution reported herein were represented as the mean ± standard deviation (SD), and variables of skewness distribution were expressed as the median value (interquartile range, IQR). Pearson's χ^2^ test or Fisher's exact were employed to compare the categorical data expressed as numbers and percentages. Comparisons between groups were performed by adopting independent samples t-test or Mann-Whitney U test. As appropriate, within-group comparisons were performed using a paired-samples t-test or Wilcoxon signed-rank test. The primary analysis consisted of a comparing the mean change in the echocardiographic parameters between the baseline values and the six-month follow-up values between the 2 treatment groups. Bivariate correlation analysis was performed to analyze the correlation between clinical variables and the change of LVMI. Multiple linear regression analysis was employed to evaluate the effect of different clinical factors on the change in LVMI. Statistical analysis was performed using the SAS software package (version 9.4, SAS Institute Inc., Cary, NC), and the statistical significance was evaluated at a two-tailed probability level of < 0.05.

## Results

### Characteristics of study subjects

**Figure 1:**
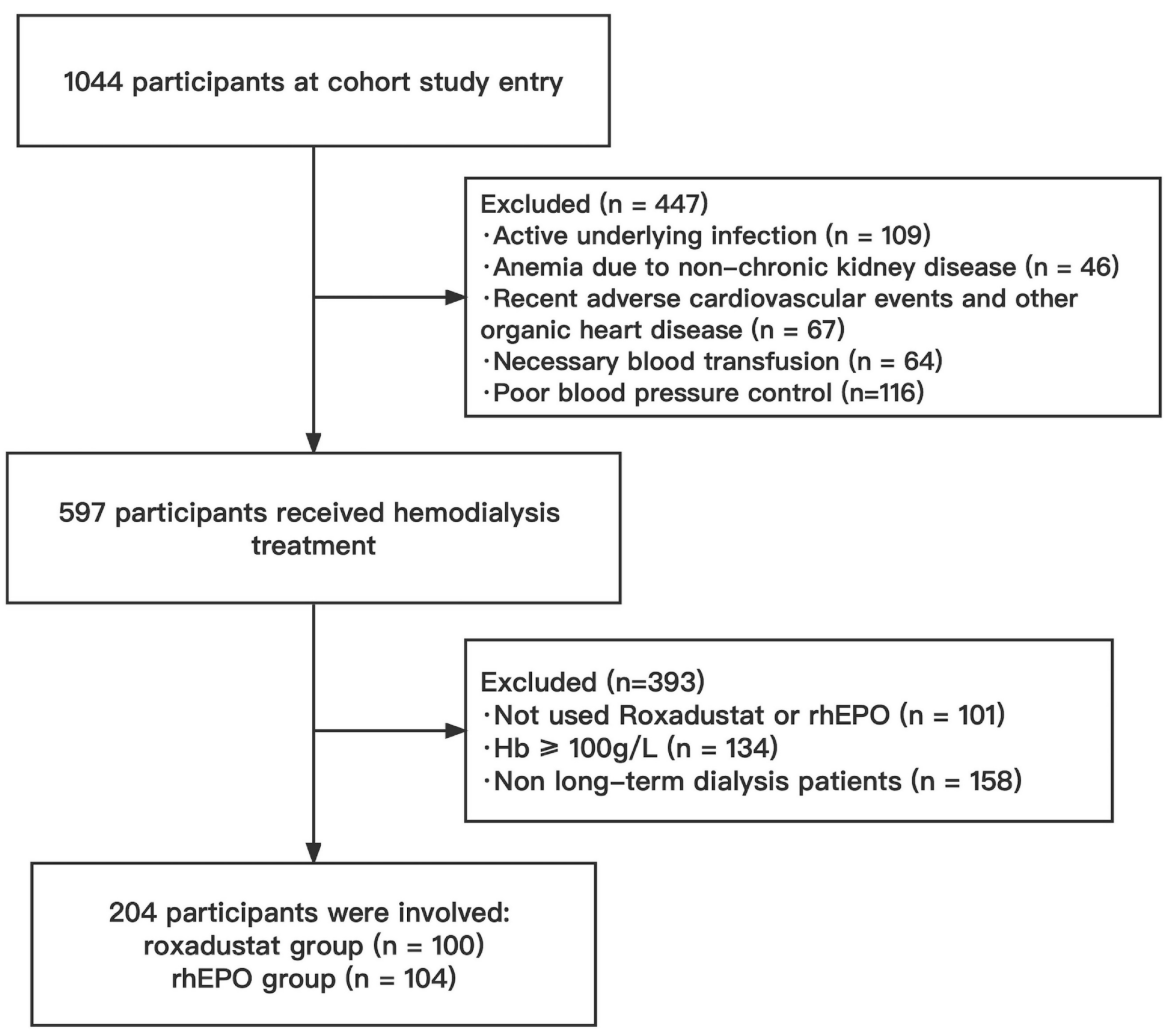
Flowchart of the study population. HD, hemodialysis; rhEPO, recombinant human erythropoietin.

1044 patients were screened from an electronic medical record database between January 2018 to October 2021. A total of 840 patients were excluded based on study inclusion and exclusion criteria (Figure 1), and the remaining eligible 204 patients were divided into two groups based on different interventions: roxadustat group (n = 100) and rhEPO group (n = 104).

Propensity score matching identified 68 patients in each cohort. The baseline characteristics of the eligible HD patients are shown in Table 1. The male participants accounted for 51.5% of the total patient population in the HD cohort. The mean ± SD age of the final HD cohort was 60 ± 14 years, and the mean ± SD body mass index (BMI) was 23.8 ± 4.2 kg/m^2^. The median duration of dialysis in all HD patients was 3 years (IQR: 2 to 5). The most common comorbidity of these patients was hypertension (89.7%), followed by diabetes mellitus (39.7%) and coronary artery disease (30.2%). Of the 136 HD patients, 82 (60.3%) were taking calcium channel blockers (CCBs), 72 (52.9%) were taking β-blockers, and 29 (21.3%) patients were taking angiotensin-converting enzyme inhibitors (ACEIs) or angiotensin receptor blockers (ARBs). After propensity score matching, there was no significant difference in the baseline clinical, laboratory, or echocardiographic characteristics between these two groups.

### Effects of Roxadustat and rhEPO on ventricular remodeling in dialysis patients

The changes in the echocardiographic outcomes of the HD population from baseline to 6-months-post treatment are summarized in Table 2. Compared to the baseline, there was a notable decrease in the LVMI of the roxadustat-treated group from 133.99 (122.47, 156.14) (baseline) to 124.43 (105.94, 148.72) g/m^2^ after the 6-month treatment duration (P < 0.001). The LVEDd and LVPWT also notably decreased from 5.16 ± 0.63 (baseline) to 4.98 ± 0.64 cm after the 6-month roxadustat treatment duration (P < 0.001) and from 1.12 ± 0.14 (baseline) to 1.08 ± 0.13 cm after the 6-month treatment duration (P < 0.001), respectively. The impact of rhEPO on LVMI was significant, decreasing from 135.39 ± 31.89 to 129.21 ± 28.08 g/m^2^ (P = 0.005), but rhEPO had no significant effect on the other echocardiographic indices. Our data showed that roxadustat was associated with obvious improvements in both SBP and DBP [from 154(138, 162) to 143(127,155) mm Hg (P < 0.001) and from 81 ± 12 to 75 ± 10 mm Hg (P = 0.001), respectively], whereas rhEPO group had no significant changes.

**Figure 2:**
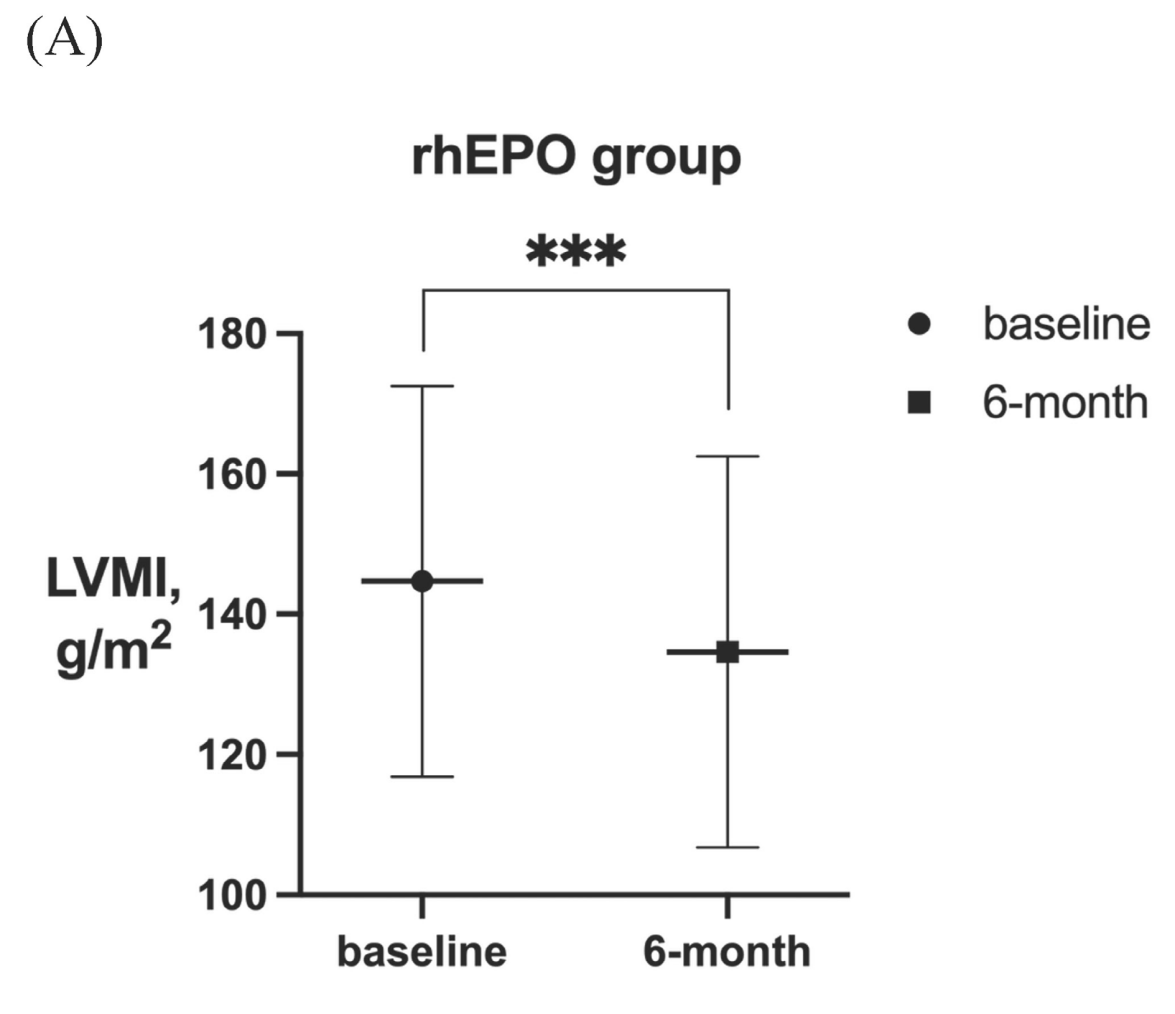

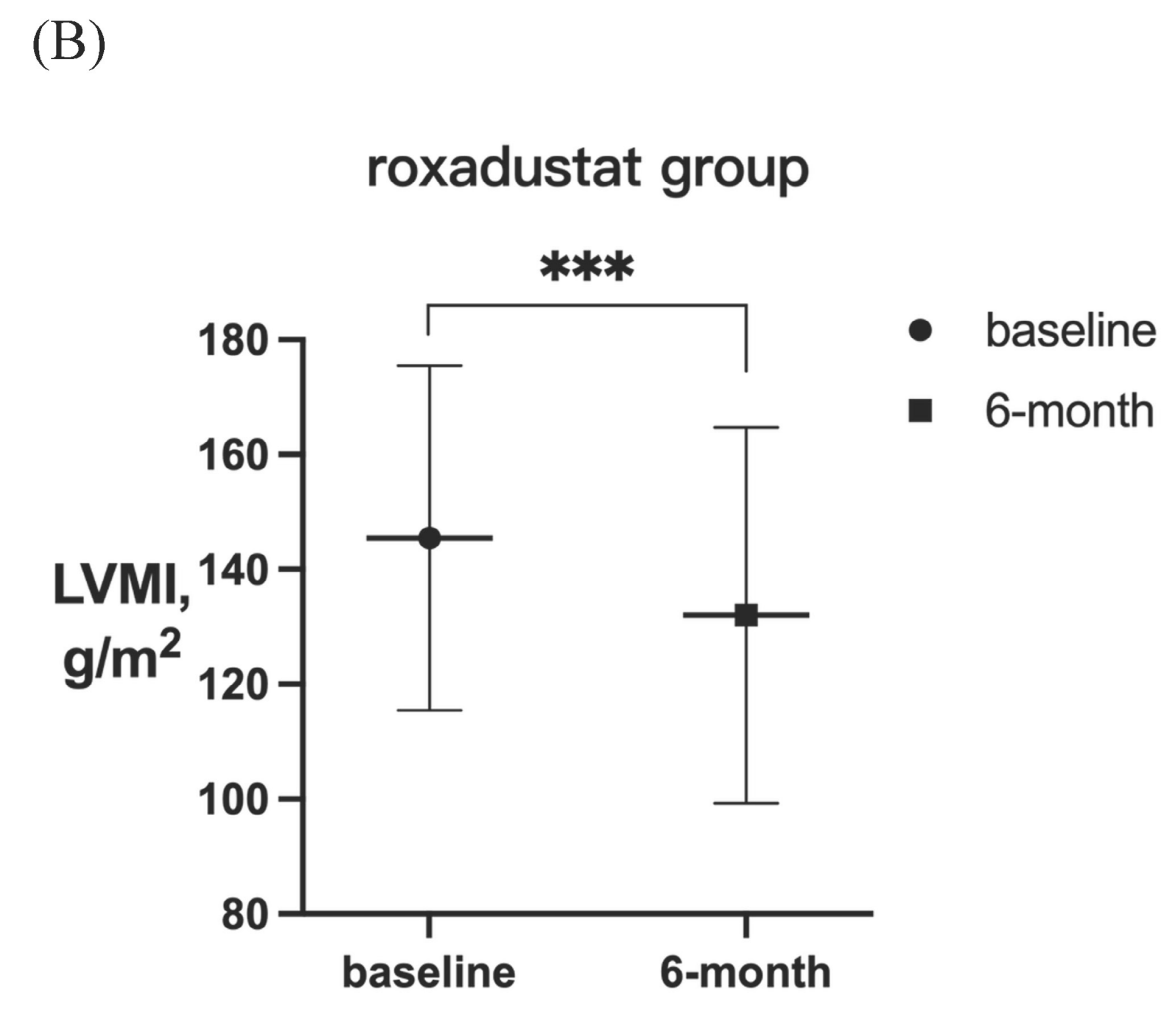

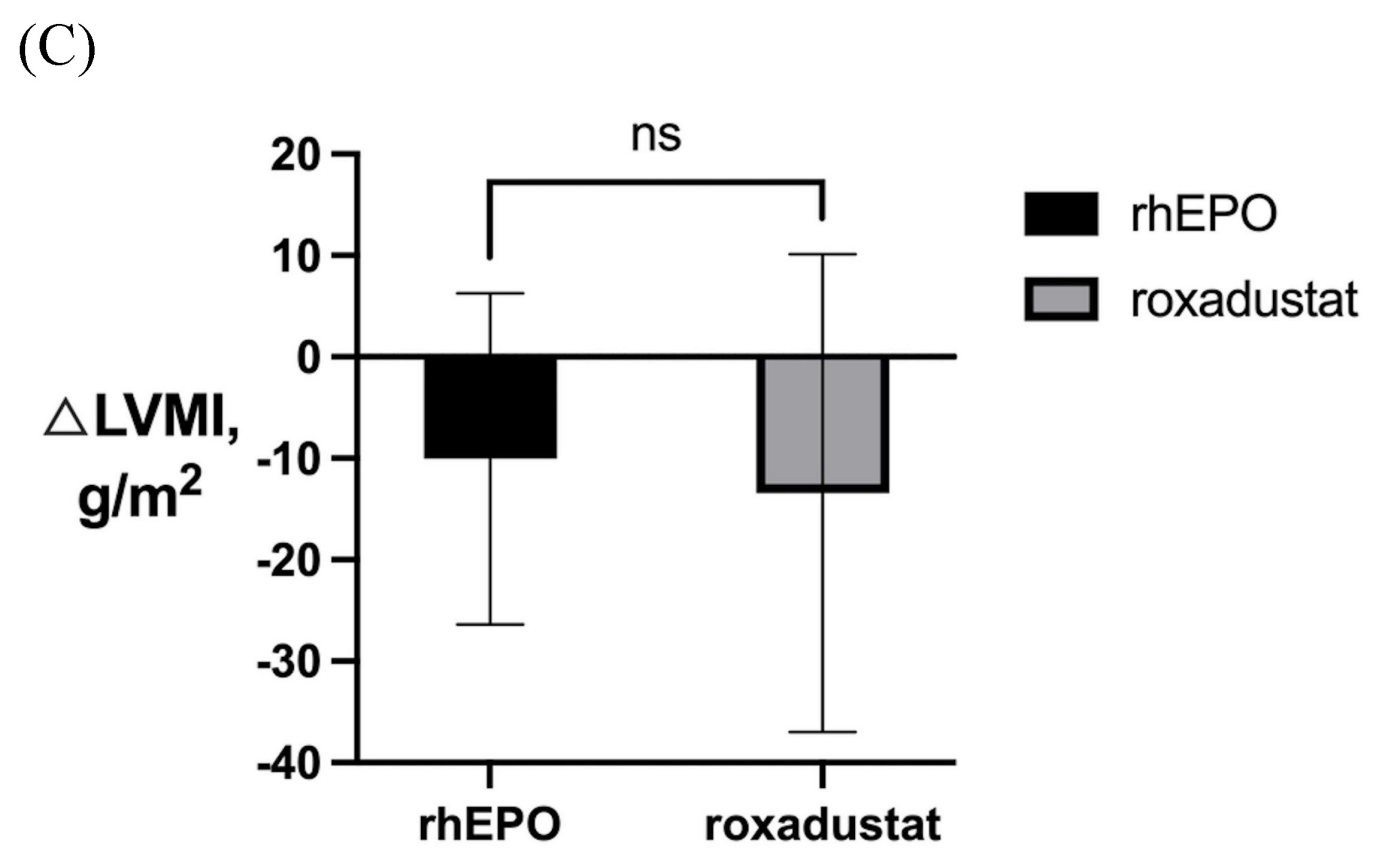
LVMI in patients with LVH before and after 6-month treatment in (A) rhEPO group (n =55) and (B) roxadustat group (n=60). (C) Change in LVMI between rhEPO group and roxadustat group. LVMI, left ventricular mass index. * P<0.033, ** P<0.002, *** P<0.001.

**Figure 3:**
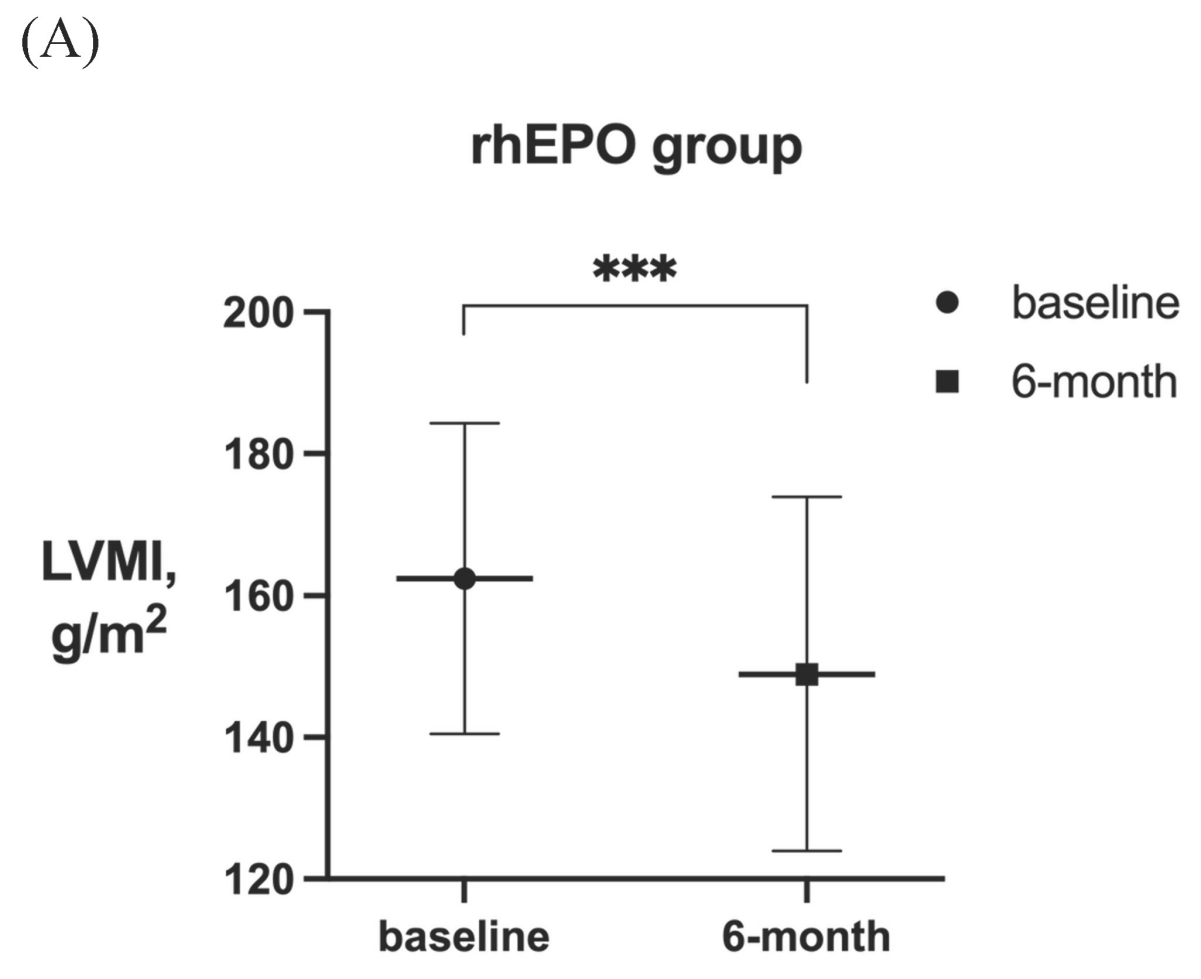

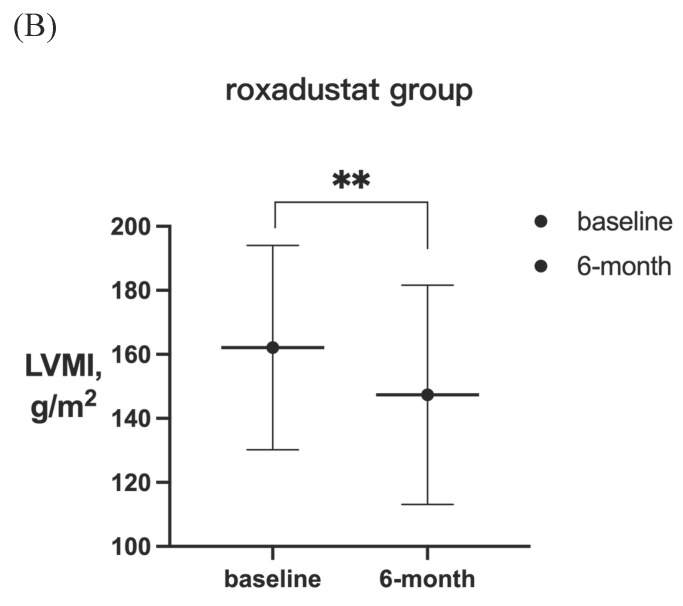

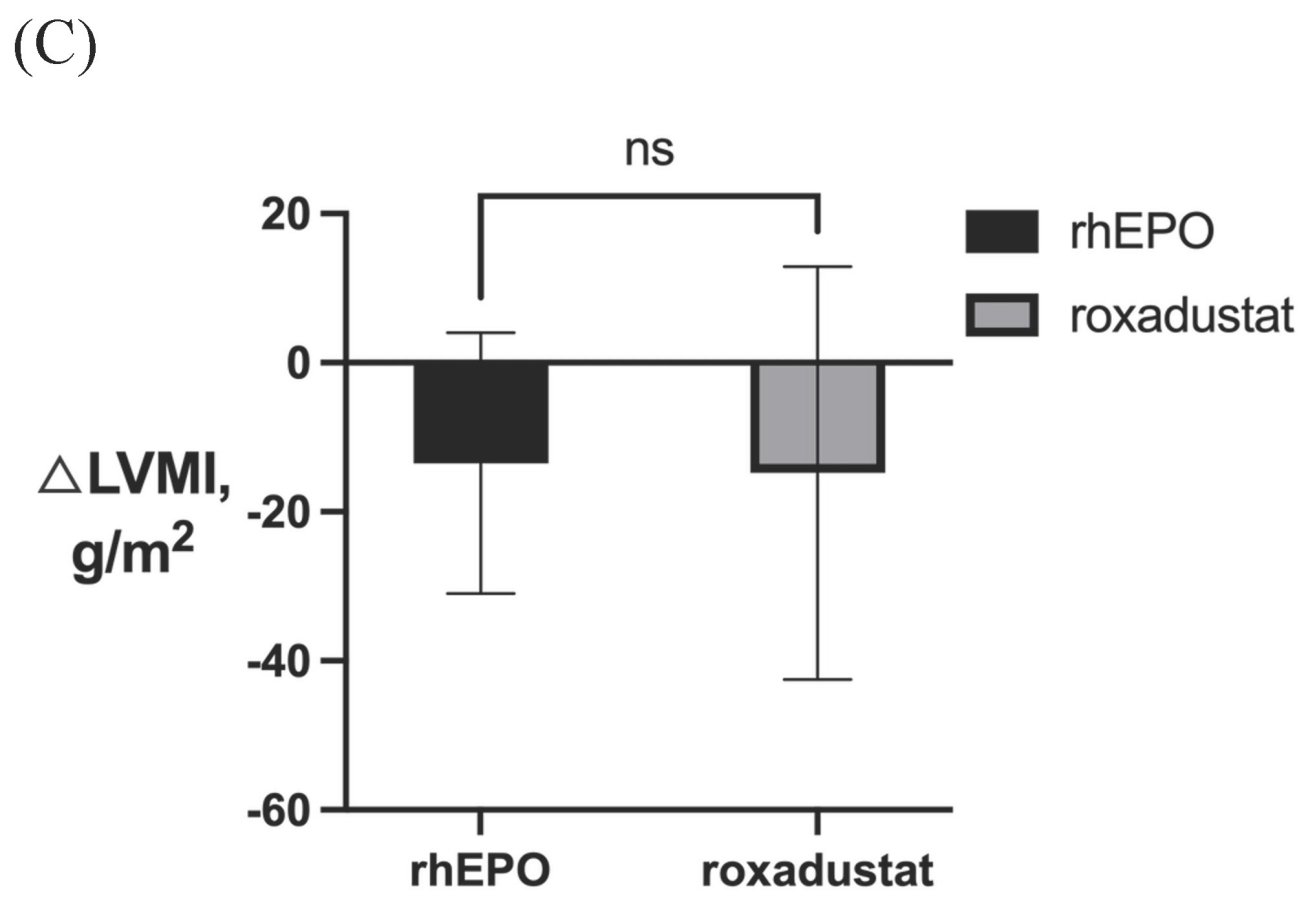
LVMI in patients with severe LVH before and after 6-month treatment in (A) rhEPO group (n=31) and (B) roxadustat group (n=31). (C) Change in LVMI between rhEPO group and roxadustat group. LVMI, left ventricular mass index. * P<0.033, ** P<0.002, *** P<0.001.

After 6-month treatment, LVMI of 60 patients with LVH in the roxadustat group decreased from 145.46 ± 30.01 g / m^2^ to 132.03 ± 32.7 g / m^2^ (P < 0.001). LVMI of 55 patients with LVH in rhEPO group decreased from 144.67 ± 27.85 g / m^2^ to 134.62 ± 27.85 g / m^2^ (P < 0.001). There was no significant difference in △LVMI (LVMI difference before and after treatment) between the two interventions in LVH patients (Figure 2). The LVMI of 31 patients with severe LVH was decreased from 162.16 ± 31.92 g/m^2^ to 147.36 ± 34.23 g/m^2^ after 6 months of roxadustat treatment (P = 0.006), and the LVMI of 31 patients with severe LVH was reduced from 162.40 ± 21.91 g/m^2^ to 148.93 ± 24.99g/m^2^ after using rhEPO (P<0.001). There was no significant difference in △LVMI between the two interventions in patients diagnosed with severe LVH (Figure 3).

Table 3 shows the magnitude of the changes in the echocardiography outcomes in dialysis population. The influences of roxadustat and rhEPO on the evaluation indices of cardiac structure and function, including the changes of LVMI, LVEDd, IVST, LVPWT, and LVEF, were not significantly different between these two groups. However, compared to rhEPO, roxadustat might result in a more significant reduction in the SBP from baseline to 6-month treatment [-12.7 ± 18.8 mm Hg (roxadustat) vs 0.9 ± 24.7 mmHg (rhEPO), P = 0.001]. There was no significant difference in DBP change between the two groups.

With changes in LVMI as dependent variables, and treatment methods (roxadustat and rhEPO), gender, age, BMI, dialysis course, cardiovascular history, cardiovascular drug history, arterio-venous fistula, changes in SBP, DBP and Hb, as independent variables, bivariate correlation analysis was conducted to analyze the correlation between various clinical variables and the changes in LVMI. Table 4a summarizes the correlation between clinical variables and reverse remodeling. Changes in LVMI were significantly negatively correlated with changes in Hb. Multiple linear regression analysis (Table 4b) showed that the change in Hb was an independent factor influencing the change in LVMI, that is, the improvement of Hb was correlated with the improvement of ventricular remodeling.

### Laboratory outcomes

The changes in laboratory indexes before and after the two treatments are shown in Table 5. Compared to baseline, statistically significant improvements in the concentrations of Hb, albumin (Alb), lipoprotein(a) [Lp(a)], unsaturated iron and total iron binding capacity (TIBC) after treatment for 6 months in both treatment groups were observed. Notably, the roxadustat group experienced a significant reduction in the levels of total cholesterol (TC) [ from 3.92(3.27, 4.50) to 3.51(2.89, 4.01) mmol/L, P = 0.01] and low-density lipoprotein (LDL) [ from 1.99(1.61, 2.68) to 1.89(1.45, 2.29) mmol/L, P = 0.014] compared to the baseline. Compared with patients treated with roxadustat, patients treated with rhEPO had a more significant increase in Alb [ 2.66 ± 4.54 mmol/L(rhEPO) vs 1.07 ± 4.6 mmol/L(roxadustat), P = 0.045]. However, roxadustat did not differ significantly from rhEPO in its effect on remaining laboratory indices after the 6-month treatments (Table 5).

## Discussion

Evidence shows that improving anaemia effectively improves cardiac structure and function^9^, ^17^, ^18^. Parameters such as LV volume and mass, among others related to cardiac structure and function, can be accurately and conveniently evaluated by echocardiography. It was previously reported that LV hypertrophy (LVH), a deleterious change in cardiac structure and function indicative of CVD, occurs in approximately 75% of ESRD patients receiving long-term dialysis^19^. Furthermore, increases in the LV volume were independently and positively related to the incidence of CVD and mortality in ESRD patients, and reductions in the LV mass were also correlated to reductions in cardiovascular events and an increased survival rate in ESRD patients 1. Other studies indicated that LVH might precede adverse cardiovascular events. Therefore, our study selected the left ventricular mass index change as the primary endpoint to assess the cardiovascular prognosis in renal anaemia patients receiving long-term dialysis.

At present, rhEPO is the most common therapeutic agent administered to treat renal anaemia because it stimulates the production of erythrocytes, and the receptors to which it binds are widely expressed in many non-erythrocyte blood cells. However, the excessive administration of rhEPO can lead to non-hematopoietic effects, such as hypertension, thrombosis, vascular remodeling, and even tumour growth. Previously, it was demonstrated that the risk of cardiovascular-related death in dialysis patients increased significantly as the dose of rhEPO increased^20^, ^21^. HIF-PHIs, including roxadustat, represent a novel class of orally available drugs for renal anaemia and can regulate the expression of hypoxia-inducible factor (HIF), promoting the production of endogenous EPO and improving iron absorption and utilization 22. However, current studies related to the clinical efficacy of HIF-PHIs have primarily focused on their effects on the improvement of anaemia and the occurrence of cardiovascular adverse events. There were no studies on the efficacy of these new drugs for improving cardiac hypertrophy (i.e., LVH) in patients with ESRD receiving long-term dialysis. Therefore, we assessed whether targeting anaemia would lead to overall improvements in cardiac structure and function by monitoring the change of echocardiographic indices in patients with renal anaemia treated with roxadustat or rhEPO for six months. We hypothesized that the renal anaemia and metabolic disorders that the patients treated in this study with two different therapeutics might be associated with cardiac dysfunction.

There is a bidirectional interaction between heart and kidney disease in acute and chronic dysfunction. From a pathophysiological perspective, CVD and CKD share many common pathways 23 , such as anaemia, microinflammation, and dyslipidemia, to name a few. In one observational study involving 432 dialysis patients, anaemia was associated with an increase in LVMI 24. Patients with renal anaemia may experience pathological changes in cardiac structure and function after long-term renal replacement therapy due to irreversible reduction of renal function, the persistence of micro-inflammatory states, oxidative stress, the activation of inflammatory pathways, and the role of the sympathetic nervous system. However, any analysis of the association between LVH and anaemia may be confused by other factors affecting the results, such as low blood pressure control and poor cardiac structure 25.

Previous studies confirmed that early-stage LVH was reversible if haemoglobin levels could be increased quickly and effectively^9^. It was consistent with previous clinical trials 12 that roxadustat and rhEPO both significantly engendered similar increases in haemoglobin levels in the studied dialysis patients. Although the results demonstrated that both drugs had a positive effect on reducing LVMI, the two drugs did not show clear superiority or inferiority in six months in this study. In addition, our paper found that the LVMI of patients diagnosed with LVH or severe LVH at baseline decreased significantly after 6-month treatments. Further analysis showed that the increase of Hb was an important factor in reversing cardiac remodeling. Therefore, our study shows that cardiac structure and functional parameters may be reversed with the improvement of anaemia. The results further strengthened the hypothesis that improving anaemia would have an overall beneficial effect on improving cardiac structure and function. However, the increase in haemoglobin level could not fully explain the changes in cardiac structure in the dialysis population, as there might be other factors unrelated to anaemia.

There are many complex factors affecting the change of LVMI in the dialysis population. Poor blood pressure control due to overload of capacity, water-sodium retention, and renin-angiotensin-aldosterone system (RAAS) sympathetic excitation are independent risk factors of LVH and cardiac remodeling from which dialysis patients suffer 26, 27. The pathogenesis of hypertension is more complex in dialysis patients than in non-dialysis patients, and the risk of cardiovascular events due to hypertension is higher in dialysis patients. Therefore, blood pressure management is integral in managing a good quality of life and a long-term cardiovascular prognosis in dialysis patients. Notably, our data showed that roxadustat was superior to rhEPO for improving SBP and DBP, suggesting that roxadustat in contrast to rhEPO, had a blood pressure lowering effect in dialysis patients. When blood pressure elevates, resulting in increased left ventricular afterload, adequate cardiac output can be maintained by enhancing myocardial contractility. However, prolonged increased cardiac afterload can lead to myocardial hypertrophy, thickening of muscle fibers, and decreased relative capillary density, ultimately promoting LVH. If hypertension is well controlled, as mentioned above, cardiac pathophysiological changes may be reversed. Therefore, roxadustat is recommended for patients with poor blood pressure control.

Concerning iron metabolism, two therapies both positively impacted serum ferritin and TIBC levels. It was previously demonstrated that HIF-PHI was different from rhEPO and intravenous iron in treating renal anaemia 28. The level of serum EPO in patients receiving HIF-PHIs was lower than that in the patients receiving rhEPO, while HIF-PHIs led to a decrease in the level of hepcidin, improving the utilization of existing iron storage and promoting the absorption of oral iron. Patients would not have to rely on the intravenous administration of high doses of iron, thereby mitigating inflammation and ultimately preventing mortality 29. However, the effect of the two drugs on iron metabolism did not appear to be significantly different in our study, and this result may need to be explored in more trials. It was worth noting that roxadustat was associated with decreased TC and LDL, while the rhEPO group had no significant effect on lipid metabolism, which confirmed the positive effect of HIF-PHIs on lipid metabolism. Current studies^30^ have shown that HIF-1 α is involved in regulating the degradation of 3-hydroxy-3-menthyl glutarate mono-acyl-CoA reductase, reducing cholesterol synthesis and regulating lipid metabolism under hypoxia. Therefore, compared with traditional ESA, roxadustat has apparent advantages in terms of mechanism, improving lipid metabolism to a greater extent. In addition, malnutrition can aggravate the degree of anaemia in hemodialysis patients, and severe anaemia further deteriorates the nutritional status of patients. In this study, the serum Alb level of the patients after treatment with both drugs was higher than before, which may be related to the improvement of the patient's appetite after the anaemia was corrected.

### Limitations

There are some limitations to this retrospective, single-centre cohort study. Firstly, as treatment is an exposure factor and a clinical decision, the administration of a particular treatment requires the consideration of a variety of confounding factors, such as the age and socioeconomic status of the patient and the severity of their disease. Although no clear difference in the effects of the two drugs on LVMI was observed in this study, it is only a 6-month assessment, and it has not been confirmed whether this difference would not occur at a 12-month assessment. Strict inclusion and exclusion criteria were developed, but there might still be selection bias in the cohorts. Secondly, the follow-up data of some patients are missing, especially data on the levels of hypersensitive C-reactive protein, interleukin-6, and tumor necrosis factor-α, which resulted in the inability to evaluate the influence of the two treatments on microinflammation indicators more comprehensively. Thirdly, although all ultrasound examinations were conducted by qualified technicians, there were also unavoidable measurement errors. Echocardiography may have some limitations in the dialysis population, given the cardiac structural abnormalities in patients receiving long-term dialysis and the changes in volume load during dialysis. Although data on the changes in left ventricular systolic volume were not acquired, LVEF can also evaluate systolic cardiac function well to some extent^31^, ^32^. The study failed to obtain clinical symptoms and adverse side effects from the electronic medical record system, so this paper did not discuss the safety of the drugs.

## Conclusion

This study is the first to compare the effects of roxadustat and rhEPO on cardiac structure and function. Although causality was not established in this observational study, extensive analyses were conducted to confirm the effects of anaemia, blood pressure management, and metabolic factors on cardiac structure and clinical outcomes. This paper demonstrated that increased haemoglobin was associated with improved left ventricular diastolic function in dialysis patients, and cardiac hypertrophy due to anaemia is reversible. Roxadustat can simultaneously improve patients' lipid and iron metabolism, with a less effect on blood pressure. Our assessment of subclinical CVD will provide a reference for the cardiovascular safety assessment of HIF-PHIs. Therefore, it is essential to conduct more extensive, multi-centre, randomized controlled trials to evaluate better the impact of roxadustat on cardiac structure and function in the future.

## Figures and Tables

**Table 1:** Baseline characteristics of the hemodialysis population

	rhEPO group(n=68)	Roxadustat group(n=68)	*P-* value
Age, yr	59±14	62±13	0.203
Male, n (%)	33(48.5)	37(54.4)	0.493
BMI, kg/m^2^	22.9(21.2, 26.7)	23.8(21.5, 25.7)	0.588
Dialysis duration, yr	3(2, 5)	3(2, 4)	0.307
Arteriovenous fistula, n (%)	51(75.0)	53(77.9)	0.686
Comorbidities, n (%)	66(97.1)	66(97.1)	1.000
Hypertension	61(89.7)	61(89.7)	1.000
Coronary artery disease	21(30.9)	20(29.4)	0.852
Atrial fibrillation	15(22.1)	13(19.1)	0.671
Diabetes mellitus	26(38.2)	28(41.2)	0.726
Cardiovascular medication, n (%)	46(67.7)	45(66.2)	0.855
ACEI/ARB	14(20.6)	15(22.1)	0.834
Beta-blocker	35(51.5)	37(54.4)	0.731
Aldosterone receptor blocker	8(11.8)	7(10.3)	0.784
ARNI	12(17.7)	11(16.2)	0.819
CCB	39(57.4)	43(63.2)	0.483
SBP, mmHg	148(128, 160)	154(138, 162)	0.095
DBP, mmHg	78(68, 86)	79 (75, 89)	0.197

HD, hemodialysis; EPO, erythropoietin; BMI, body mass index; SBP, systolic blood pressure; DBP, diastolic blood pressure; ACEI, angiotensin-converting enzyme inhibitor; ARB, angiotensin receptor blocker; ARNI, angiotensin receptor neprilysin inhibitor; Data are presented as mean ± SD or median (interquartile range).

**Table 2:** Changes in left ventricular parameters and blood pressure after 6 month or treatment

	rhEPO group (n=68)			roxadustat group (n=68)		
	baseline	6-month	*P* value	baseline	6-month	*P* value
IVST, cm	1.18 ± 0.18	1.18 ± 0.19	0.744	1.20(1.07, 1.30)	1.20(1.03, 1.25)	0.319
LVEDd, cm	5.06±0.62	4.97±0.61	0.080	5.16±0.63	4.98±0.64	<0.001
LVPWT, cm	1.10(0.98, 1.20)	1.10(0.97, 1.20)	0.318	1.12±0.14	1.08±0.13	<0.001
LVEF	0.66(0.61, 0.74)	0.69(0.59, 0.73)	0.408	0.67(0.61, 0.73)	0.69(0.60, 0.72)	0.676
LVMI, g/m^2^	135.39 ± 31.89	129.21 ± 28.08	0.005	133.99(122.47, 156.14)	124.43(105.94, 148.72)	<0.001
SBP, mmHg	148(128, 160)	141(128, 160)	0.806	154(138, 162)	143(127, 155)	<0.001
DBP, mmHg	78(68, 86)	78(68, 85)	0.768	81 ± 12	75 ± 10	0.001

IVST, interventricular septal thickness; LVEDd, left ventricular end-diastolic diameter; LVPWT, left ventricular posterior wall thickness; LVEF, left ventricular ejection fraction; LVMI, left ventricular mass index.

**Table 3:** Changes in parameters measured by Echocardiography after 6 months treatment

	rhEPO group (n= 68)	roxadustat group (n= 68)	*P* value
△IVST, cm	-0.01 ± 0.15	-0.02 ± 0.17	0.530
△LVEDd, cm	-0.09 ± 0.43	-0.19 ± 0.45	0.224
△LVPWT, cm	-0.02 ± 0.16	-0.03 ± 0.15	0.533
△LVEF	0.01 ± 0.09	-0.01 ± 0.08	0.257
△LVMI, g/m^2^	-6.98(-16.81, 6.99)	-8.86(-24.24, 3.58)	0.297
△SBP, mmHg	-1 ± 24	-11 ± 19	0.011
△DBP, mmHg	-1 ± 16	-5 ± 12	0.078

**Table 4a:** Relationships between changes in LVMI and clinical parameters

	Correlation coefficient	P
Treatment	-0.132	0.126
Age, yr	-0.063	0.467
BMI, g/m^2^	-0.041	0.64
Dialysis course, yr	0.043	0.623
Cardiovascular history	-0.032	0.715
Cardiovascular medications	-0.027	0.753
Arterio-venous fistula	-0.107	0.213
△SBP, mmHg	0.004	0.966
△DBP, mmHg	0.097	0.262
△Hb, g/L	-.0268	0.002

**Table 4b:** Relationships between changes in LVMI and clinical parameters in the hemodialysis population according to multiple linear regression analysis

	Regression coefficients	95.0% CI		P
(Constant)	11.689	-20.169	43.548	0.469
Treatment	-4.721	-11.74	2.298	0.186
Age, yr	-0.052	-0.325	0.221	0.709
BMI, g/m^2^	0.065	-0.789	0.92	0.88
Dialysis course, yr	0.272	-1.338	1.881	0.739
Cardiovascular history	-6.086	-27.129	14.957	0.568
Cardiovascular medications	-3.508	-11.189	4.173	0.368
Arterio-venous fistula	-5.918	-14.296	2.46	0.165
△SBP, mmHg	-0.137	-0.34	0.066	0.185
△DBP, mmHg	0.261	-0.06	0.582	0.11
△Hb, g/L	-0.299	-0.487	-0.112	0.002

95% CI, 95% confidence interval.

**Table 5:** Laboratory parameters at baseline and 6 months

	rhEPO group (n=68)			roxadustat group (n=68)		
	baseline	6-month	*P* value	baseline	6-month	*P* value
Hb, g/L	91(82, 95)	103(95, 112)	<0.001	83(73.50, 93.75)	99(88, 111)	<0.001
Alb, mmol/L	35.51 ± 5.65	38.17 ± 4.61	<0.001	33.38 ± 4.99	34.45 ± 5.29	0.001
TG, mmol/L	1.43(0.95, 1.97)	1.32(0.96, 2.04)	0.837	1.55(1.11, 2.26)	1.47(1.11, 1.83)	0.364
TC, mmol/L	4.09 ± 1.06	4.06 ± 1.28	0.822	3.92(3.27, 4.50)	3.51(2.89, 4.01)	0.010
HDL, mmol/L	1.14(0.87, 1.33)	1.15(0.95, 1.34)	0.222	1.05(0.85, 1.30)	0.95(0.80, 1.24)	0.077
LDL, mmol/L	2.31 ± 0.77	2.26 ± 0.93	0.651	1.99(1.61, 2.68)	1.89(1.45, 2.29)	0.014
Lp(a), mg/L	236(95, 413)	186(65, 299)	0.011	228(107, 445)	195(75, 391)	0.005
Uric acid, μmol/L	345.6 ± 120.2	351.3 ± 111.3	0.691	410.0(325.3, 468.0)	366.9(307.5, 449.8)	0.460
PTH, pg/mL	218.6(94.0, 431.3)	229.8(113.4, 499.5)	0.319	205.9(102.1, 403.7)	191.0(84.4, 349.6)	0.935
SF, ug/dL	132(71.58, 258.92)	82.25(45.46, 186.25)	0.001	100.11(53.03, 245.02)	77.1(44.27, 206.93)	0.004
EPO, mIU/mL	10.30(8.21, 36.90)	16.91(7.51, 48.77)	0.263	8.89(5.88, 18.68)	13.21(7.43, 35.03)	0.158
SI, ug/dL	8.64(6.70, 12.01)	8.8(5.38, 11.75)	0.559	8.31(6.11, 10.10)	8.8(5.60, 12.86)	0.209
TIBC, ug/dL	37.72(27.51, 47.12)	46.36(35.67, 57.76)	0.001	43.7(29.15, 55.74)	51.19(44.35, 60.10)	<0.001
Unsaturated iron, ug/dL	28.94(18.04, 33.50)	38.81(24.36, 48.80)	<0.001	32.71(23.41, 48.08)	41.35(30.26, 50.57)	0.002

Hb, haemoglobin; Alb, albumin; TG, total triglycerides; TC, total cholesterol; HDL, high-density lipoprotein; LDL, low-density lipoprotein; Lp(a), lipoprotein(a); SF, serum ferritin; SI, serum iron; TIBC, total iron binding capacity.

## Abbreviations

CKD: chronic kidney disease
CVD: cardiovascular disease
LVH: left ventricular hypertrophy
ESRD: end-stage renal disease
Hb: haemoglobin
ESA: erythrocyte-forming stimulants
LVMI: left ventricular mass index
HIF-PHIs: hypoxia-inducible factor prolyl hydroxylase inhibitors
HD: haemodialysis
rhEPO: recombinant human erythropoietin
STROBE: the Strengthening the Reporting of Observational Studies in Epidemiology
KDIGO: Kidney Disease - Improving Global Outcomes
SBP: systolic blood pressure
DBP: diastolic blood pressure
ACC/AHA: American College of Cardiology/American Heart Association
left ventricular: LV
ASE: American Society of Echocardiography
LVEF: left ventricular ejection fraction
LVEDd: left ventricular end-diastolic diameters
IVST: interventricular septal thickness
LVPWT: left ventricular wall thickness
SD: standard deviation
IQR: interquartile range
BMI: body mass index
CCBs: calcium channel blockers
ACEI: angiotensin-converting enzyme inhibitors
ARBs: angiotensin receptor blockers
Alb: albumin
TG: total triglycerides
TC: total cholesterol
HDL: high-density lipoprotein
LDL: low-density lipoprotein
Lp(a): lipoprotein(a)
SF: serum ferritin
SI: serum iron
TIBC: total iron binding capacity
RAAS: renin-angiotensin-aldosterone system
